# Experimental Estimating Deflection of a Simple Beam Bridge Model Using Grating Eddy Current Sensors

**DOI:** 10.3390/s120809987

**Published:** 2012-07-25

**Authors:** Chunfeng Lü, Weiwen Liu, Yongjie Zhang, Hui Zhao

**Affiliations:** Department of Instrument Science and Engineering, Shanghai Jiao Tong University, Shanghai 200240, China; E-Mails: chunfenglv@sjtu.edu.cn (C.L.); weiwenliu@sjtu.edu.cn (W.L.); zhangyongjie@sjtu.edu.cn (Y.Z.)

**Keywords:** three-point method, deflection estimation, relative deflection, absolute deflection, grating eddy current sensor (GECS)

## Abstract

A novel three-point method using a grating eddy current absolute position sensor (GECS) for bridge deflection estimation is proposed in this paper. Real spatial positions of the measuring points along the span axis are directly used as relative reference points of each other rather than using any other auxiliary static reference points for measuring devices in a conventional method. Every three adjacent measuring points are defined as a measuring unit and a straight connecting bar with a GECS fixed on the center section of it links the two endpoints. In each measuring unit, the displacement of the mid-measuring point relative to the connecting bar measured by the GECS is defined as the relative deflection. Absolute deflections of each measuring point can be calculated from the relative deflections of all the measuring units directly without any correcting approaches. Principles of the three-point method and displacement measurement of the GECS are introduced in detail. Both static and dynamic experiments have been carried out on a simple beam bridge model, which demonstrate that the three-point deflection estimation method using the GECS is effective and offers a reliable way for bridge deflection estimation, especially for long-term monitoring.

## 1. Introduction

After a bridge is put into use, gradual deterioration is inevitable because of loading, temperature changes or other environmental factors. In order to guarantee the safety and durability of those bridges which are expensive and closely related with people's livelihood, long-term and continuous structural health monitoring is an essential part of the maintenance management. Among the various structural performance evaluations, vertical deflection is an important parameter that can directly and effectively indicate a bridge's behavior.

In terms of instrumentation for deflection estimation, there are contact and non-contact deflection estimation methods. Traditional displacement sensors such as mechanical dial gauges or linear variable differential transducers (LVDTs) are used in contact measurement, through which static or real-time displacement values can be obtained directly or fed into a computer for processing and displaying via a data cable. This method, however, requires access under the bridge and installation of a temporary supporting system to mount sensors, which is time consuming and not very efficienct. In addition, it might even be unavailable when bridges are over rivers, highways or have high clearance. Another contact sensor is the fiber optic Bragg-grating (FBG) sensor through which the deflection is calculated from the measured strain data and displacement-strain relationship [1,2]. In this way, however, the calculated displacement from strain data is sensitive to noise, and the sensors are expensive and must be embedded into the structure, which to a certain degree is difficult for bridges in service.

To cope with those inconveniences in contact measuring methods, various non-contact approaches have been proposed. Based on the detection of the Doppler shift of the laser light, a laser Doppler vibrometer (LDV) equipped with displacement and velocity signal decoders can measure both bridge deflection and vibration simultaneously [3]. In this way, a static reference point (usually underneath the bridge) is needed for device mounting, and the device should be attended, which limits it's usability for long-term monitoring. Among image methods, dynamic deflection with high resolution of the bridge can be obtained through using digital image processing techniques [4], while deflection distribution from the images of the bridge girder surface recorded by a digital camera before and after deformation can be evaluated by digital image correlation techniques [5], and digital close-range terrestrial photogrammetry (DCRTP) can measure the spatial coordination in three-dimensions [6,7]. Like the LDV, devices such as video cameras used in image methods cannot be left unattended and they are easily affected by weather conditions. Use of a Global Positioning System (GPS) can provide spatial locations of the measuring points on the bridge in real-time by comparing with a continuing operational reference station (CROS). It offers a long-term monitoring approach without being affected by climatic factors [8,9], but due to its relatively low accuracy, it is only applied to those bridges with significant deformations. All the non-contact methods mentioned above, although they differ in instrumentation, have one thing in common, a static reference point or CROS that is kept a certain distance away from the bridge is selected for installation of the measuring device, otherwise measurements cannot be carried out. Another method is using inclinometers which can be installed on the bridge directly along a line paralleling the bridge span axis [10,11], and both static and dynamic deflection time history curves can be calculated through curve-fitting technology based on the accurate angle records of the inclinometers. An outstanding feature of the inclinometer is that static reference points mentioned above are no longer needed. This approach reduces the dependence on environmental conditions and it is suitable for long-term monitoring.

To avoid those deficiencies in conventional estimating deflection methods mentioned above, a novel three-point deflection estimation method is presented in this paper. Measuring points along lines paralleling the bridge span axis are chosen equidistantly. Among these measuring points, every three adjacent measuring points are defined as a measuring unit in which a straight connecting bar linking the two endpoints is taken as a relative reference line. Relative deflection of the mid-measuring point relative to the intermediate point of the connecting bar on which a displacement sensor is fixed can be measured, and thus the absolute deflection of each measuring point can be calculated from the relative deflections of all the measuring units. Compared with the contact and non-contact methods mentioned above, only real spatial positions of the measuring points are taken as relative references without any other static reference points. Moreover, the selected displacement sensor is the grating eddy current absolute position sensor (GECS) which is different from traditional eddy current sensors based on vertical characteristics [12,13]. Since the structure of grating reflective conductors is adopted, the measurement range is extended but without compromising the accuracy. In addition, as an inductive sensor, the GECS is waterproof and dustproof in principle, thus it can work under bad weather conditions, which makes it ideal for long-term monitoring.

In this paper, both the principles of the three-point method and displacement measurement of the GECS are presented. Then, this three-point method for deflection estimation is verified in a simply supported girder bridge model in the laboratory. Comprehensive static and dynamic experiment results on the laboratory tests demonstrate this method is effective and offers an alternative way for bridge deflection estimation.

## 2. Principle of the Three-Point Deflection Estimation Method

**Figure 1.**
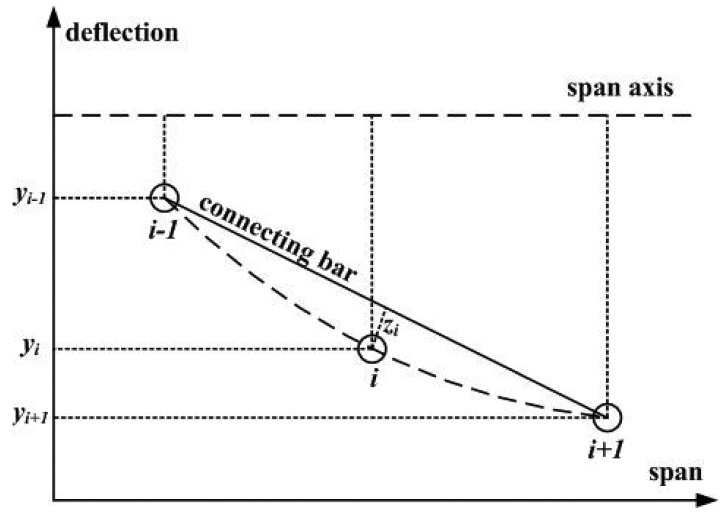
Schematic diagram of the three-point method.

The schematic diagram of the three-point method is shown in Figure 1. The absolute deflections of measuring point *i* and the two adjacent measuring points *i* − 1 and *i* + 1 are defined as *y_i_, y_i_*_-1_ and *y_i_*_+1_, respectively. A straight connecting bar is used to link the measuring points *i* − 1 and *i* + 1, and a displacement sensor is installed on it corresponding to the measuring point *i*. When deformation occurs, the absolute deflection of each measuring point relative to the span axis is different in value, and meanwhile, the relative position between the measuring point *i* and the connecting bar will be changed along the deflection direction. Therefore, the displacement can be measured by the displacement sensor installed on the connecting bar. The obtained displacement value is named as the relative deflection *z_i_*. It is to be noted that there is an angular deviation between *z_i_* and *y_i_*, but this can be ignored because it is a very slight angle deflection (often less than 2 min of angle). Thus, the absolute deflection *y_i_* of the measuring point *i* can be calculated from *y_i_*_-1_, *y_i_*_+1_ and *z_i_* (*i* = 1, 2, …, *n* − 2, *n* − 1), the relational expression of *y_i_* and *z_i_* is as follows:

(1) $$y_{i}=k_{i}(y_{i-1}+y_{i+1})+z_{i}$$

where *k_i_* is the proportionality coefficient which is related to the distance between the measuring points, especially when *k_i_* = 1/2, the measuring points are selected equidistantly.

The three-point deflection estimation method is elaborated on a laboratory simple beam bridge model, on which deflection estimation is accomplished by making use of the real spatial positions of the measuring points for relative reference points of each other rather than using any other auxiliary static reference points. Suppose the length of the beam is *L* and a total of *n* + 1 measuring points marked 0, 1, …, *n* − 1, *n* from the left to the right endpoint of the beam along lines paralleling to the span axis are chosen equidistantly. Every three adjacent measuring points are defined as a measuring unit, thus there are *n* − 1 measuring units for *n* + 1 measuring points. In each measuring unit, a straight connecting bar with the length of 2*L/n* installed on the beam linking the two endpoints of the three adjacent measuring points is used as a reference line. The displacement sensor installed on the connecting bar is the GECS which consists of a measuring board and reflective conductors and they are installed on the connecting bar and corresponding measuring point on the beam, respectively. The displacement measurement principle of the GECS will be introduced in the next section. When flexural deflection occurs, in each measuring unit, the relative position of the reflective conductors and the coils of the GECS will be changed, thus the relative deflection of each measuring unit measured by the GECS can be obtained. Therefore, the absolute deflection of all the measuring point can be calculated directly from these measured values.

**Figure 2.**
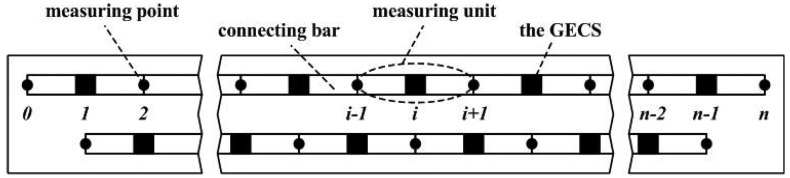
Instrumentation plan of a simple beam.

Due to the mid-measuring point in one measuring unit is used for installation of the GECS and meanwhile, it is the endpoint of the previous or next measuring unit used for connecting bar installation, which makes choice of the measuring points and connecting bars installation should be along two lines paralleled to the span axis, respectively. A complete instrumentation plan is shown in Figure 2. An individual bar is used for each measuring unit. In order to remain the connecting bar straight after the beam is loaded, the bars are free to rotate at the one end and pin-connected at the other end. A free space is reserved at the pin-connected section of the connecting bar, and it can also remove the effect of differentiate thermal deformations when the reference bar and the bridge use different materials. The beam is divided into *n* − 1 continuous measuring units, of which the relative deflection *z_i_* (*i* = 1, 2, …, *n* − 2, *n* − 1) can be obtained simultaneously while a flexural deflection happens.

The matrix expressions of *y_i_* and *z_i_* for all measuring units according to Equation (1) are as follows:

(2) Z=KY

(3) $$Y=K^{-1}Z$$

where *Z* = [*z*_1_, *z*_2_, …, *z*_n-2_, *z*_n-1_]*^T^, Y* = [*y*_1_, *y*_2_, …, *y*_n-2_, *y*_n-1_]*^T^*.

In this paper, the measuring points are chosen equidistantly, thus *k_i_* = 1/2 and the coefficient square matrix *K* is as follows and the order of it is *N* − 1:

$$K=\left[\begin{array}{ccccccc} 1 & -\frac{1}{2} & & & & & \\ -\frac{1}{2} & 1 & -\frac{1}{2} & & & 0 & \\ & & \ddots & \ddots & \ddots & & \\ & & -\frac{1}{2} & 1 & -\frac{1}{2} & & \\ & & & \ddots & \ddots & \ddots & \\ & 0 & & & -\frac{1}{2} & 1 & -\frac{1}{2}\\ & & & & & -\frac{1}{2} & 1 \end{array}\right]$$

Therefore, the absolute deflection *Y* of these *n* − 1 measuring points can be calculated from Equation (3) with the boundary conditions *y*_0_ = *y_n_* = 0 when the relative deflection estimations of all the measuring units are completed.

Furthermore, the deflection estimating is not only limited in those selected measuring point, which means one or more auxiliary measuring points can also be selected according to actual requirements. In each measuring unit, more displacement sensors can be installed on the connecting bar corresponding to the auxiliary measuring points, and from Equation (1), the absolute deflection of the auxiliary measuring point can be calculated directly with the different values of proportionality coefficient *k_i_*.

## 3. Displacement Measurement Principle of the GECS

As mentioned earlier, since the relative deflection of each measuring unit is actual an absolute displacement value measured by the GECS, from which absolute deflection of each measuring point can be calculated directly without any correcting approaches. In addition, the GECS is not susceptible to the velocity of displacement variation, and therefore, both static and dynamic deflection estimations can be realized. The selected GECS consists of a measuring board and reflective conductors. The measuring board fixed on the intermediate point of the connecting bar vertically includes four planar coils, measuring circuit and other functional modules. Coils, measuring circuit and reflective conductors can all be fabricated by the printed circuit board (PCB) technique. Grating reflective conductors usually called measuring track are two parallel series of equidistributed reflective conductors of which the spacing distance named measuring wavelength is *λ* = 5 mm and the width of each conductor along the displacement direction is *λ*/2, which is fixed on the measuring points of the beam vertically [14].

**Figure 3.**
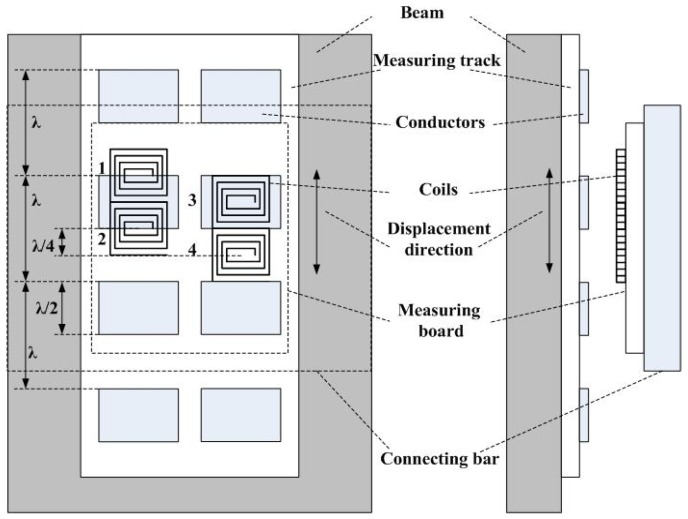
Schematic diagram of the GECS.

As shown in Figure 3, four coils marked 1, 2, 3 and 4 are divided into two parallel sets. The width of each coil is equal to that of the conductor, and the two sets of coils are *λ*/4 apart along the displacement direction. In each set, the two coils are half of the measuring wavelength apart, which forms the differential output signal to enhance the sensitivity and reduce the non-linear error. When displacement occurs, the eddy current effect between the coils and conductors will change periodically, which makes the inductance variation of each coil also periodic. Inductance of the coil can be converted into voltage, current or frequency by signal conversion circuit. In this paper, the adopted conversion circuit is frequency modulated circuit. This type of conversion circuit not only simplifies the circuit organization but also reduces mutual interference of the coils since the time sharing connection method by using the analog multiplexer.

The measuring board mainly consists of four parts:

1. Signal conversion circuit: converts inductance of the coils into electrical signal. Through an analog multiplexer, the four coils are connected to a LC oscillator circuit in turn, and outputs sinusoidal signal.
2. Signal acquisition circuit: output signals of the conversion circuit and fundamental frequency signal produced by a 12 MHz oscillator are connected to the two multi-cycle synchronous counters built-in the MCU respectively for frequency measurement and displacement calculation.
3. Power management unit: the conversion circuit and signal acquisition circuit are powered by LDO combined with the MCU through intermittent power supply mode.
4. MCU and its interface circuits: transmit the displacement value to PC or other data display modules.

Suppose the frequencies corresponding to the four coils are *f*_1_, *f*_2_, *f*_3_ and *f*_4_, the differential frequencies of these two sets of coils are *f*_12_ = *f*_1_ − *f*_2_ and *f*_34_ = *f*_3_ − *f*_4_ respectively. These two differential frequency curves approach to the characteristic of sin or cosine curves, thus the differential frequencies can be presented by the following expressions:

(4) $$f_{12}=A\sin(\frac{2\pi }{\lambda }x)$$

where *A* is the amplitude of the differential frequency, *λ* is measuring wavelength and *x* is displacement value, respectively.

Since there is a distance of *λ*/4 between these two sets along the moving direction, there is a phase separation of *π*/2 compared to *f*_12_, thus *f*_34_ is:

(5) $$f_{34}=A\cos(\frac{2\pi }{\lambda }x)$$

Suppose the phase is $\varphi (x)=\frac{2\pi }{\lambda }x$ and it can be calculated from the following expression:

(6) $$\varphi (x)=\arctan(\frac{f_{12}}{f_{34}})$$

**Figure 4.**
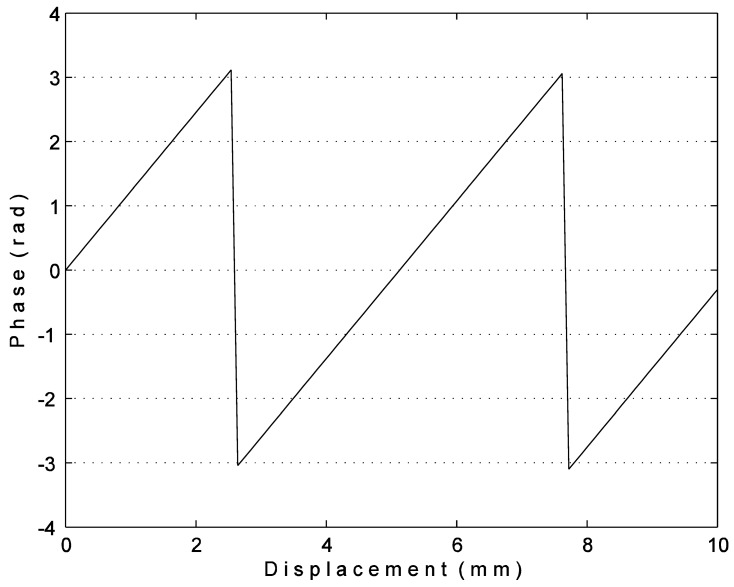
Relation curve of phase and displacement.

The phase curve of two measuring wavelength is shown in Figure 4. It can be seen that the phase and displacement are linear relation in one measuring wavelength and the displacement *x* can be obtained by:

(7) $$x=\frac{\lambda \varphi }{2\pi }$$

The accuracy of this sensor is ±0.01 mm within a measurement range which is equal to the wavelength *λ* = 5 mm, and the measurement range can be extended by increasing the measuring wavelength properly or adding another track with different measuring wavelength, e.g., *λ*_2_ = 4.88 mm. Applications of the GECS can be continually extended in other fields such as digital caliper [15], liquid level gauge and so on.

## 4. Experimental Verification of the Deflection Estimation Method

**Figure 5.**
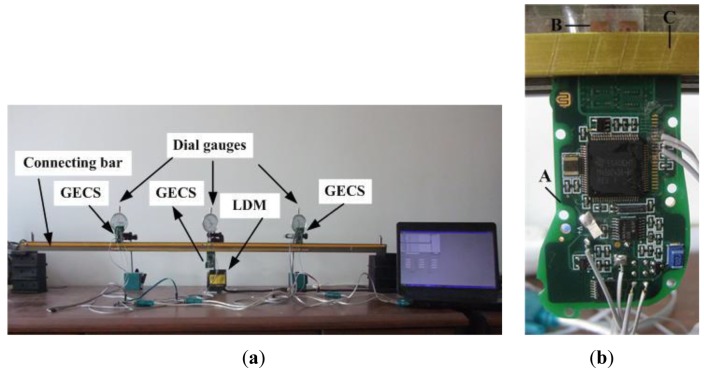
Laboratory simply beam bridge model: (**a**) deflection estimation system. (**b**) the GECS: A, B and C are the measuring board, reflective conductors and connecting bar.

A laboratory simply bridge model is shown in Figure 5. The beam is 1,280 mm long, on which the choice of the equidistant measuring points and connecting bar installation are accomplished along two lines paralleling to the span axis respectively according to Figure 2. For this model, measuring points are chosen every 320 mm along the span axis, thus five measuring points including the two endpoints are selected. The whole deflection estimation system is divided into three measuring units, and in each unit, the connecting bar is 640 mm long. Three dial gauges are used for direct reading out the absolute deflections of measuring points 1, 2 and 3, and meanwhile, a laser displacement meter (LDM) under the beam is used for recording real-time deflection of measuring point 2. For verification of the three-point method, both static and dynamic experiments have been carried out on this model.

### 4.1. Static Experimental Verification

**Figure 6.**
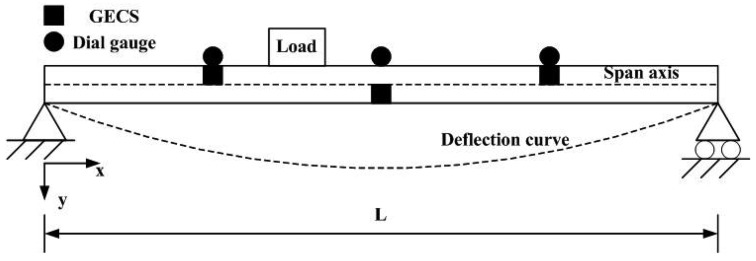
Diagram of static experiment.

Figure 6 shows the simplified deflection estimation diagram of the static experiment. During static experiments, static loads with different weights are placed on the beam. The relative deflection of each measuring unit estimated by the GECS is recorded and then the absolute deflection of the corresponding measuring points can be calculated. Table 1 lists the relative deflection estimated by the GECS of each measuring unit and the calculated absolute deflections of the corresponding measuring points. Moreover, absolute deflections measured by dial gauges for comparison and relative errors are also listed in it.

**Figure 7.**
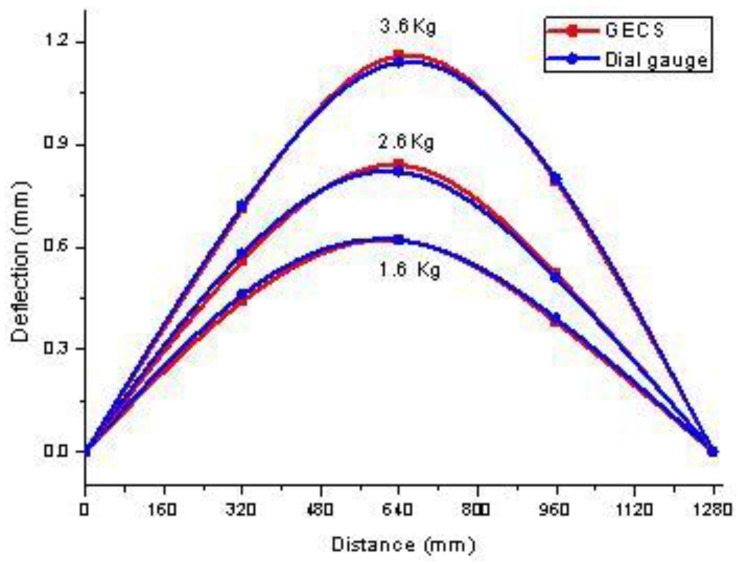
Deflection curves in different loads.

The estimated deflection curves of static loads with different weight is shown in Figure 7, and meanwhile, deflection curves estimate by dial gauges directly are used for comparisons. From these measuring results, a good agreement is shown between the results of the proposed method and that of the dial gauges.

Multiple estimating results of which loads with various weights are placed at different positions show that the deflection estimated by the three-point method is credible and the relative errors are less than 5%.

### 4.2. Dynamic Experiments Verification

While in dynamic estimations, dynamic estimated data is recorded in a time history and then it will be analyzed in time or frequency domain, which will provide the calculation basis for subsequent engineering design. The choice of the estimated data type should take the dynamic response of the object model into account. For bridge models, due to the low acceleration value and low natural frequency (often within 0.05 Hz−2 Hz), the priority estimating object selection is the displacement estimation. In this paper, two dynamic experiments include vehicle moving tests and vibration tests are performed.

**Figure 8.**
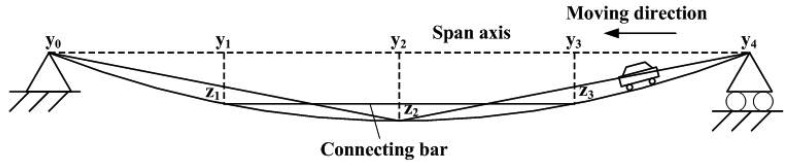
Diagram of simulated vehicle moving test.

**Figure 9.**
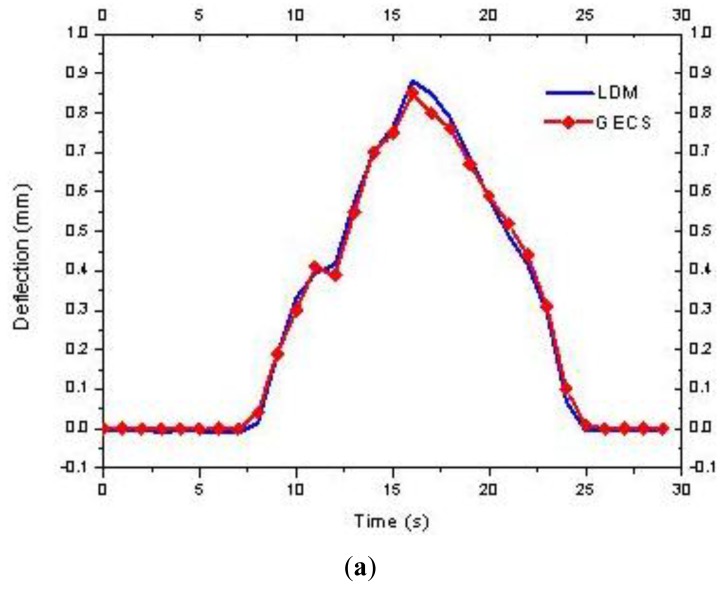

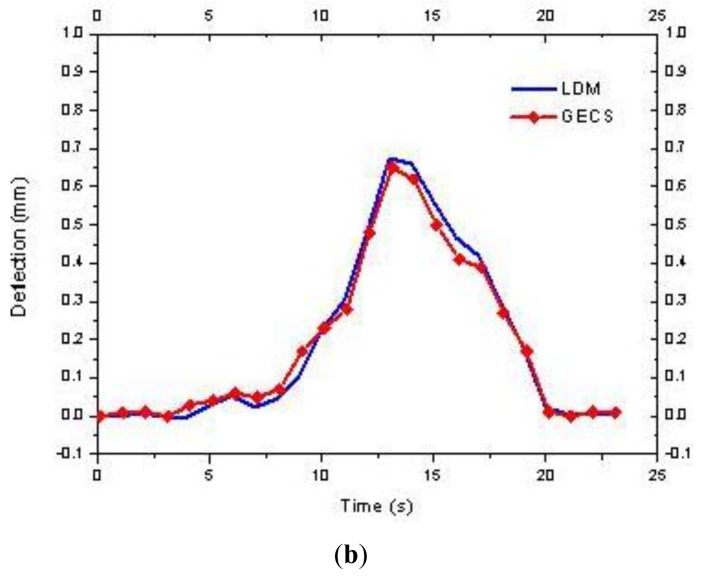
Results of simulated vehicle moving test: (**a**) v = 60 mm/s, (**b**) v = 100 mm/s.

In the simulated vehicle moving tests, time history curves of the dynamic deflection under different running speed are estimated for further analyses. During the laboratory tests, a moving load simulated the vehicle running in different speed is developed on the beam. As shown in Figure 8, the moving direction is from the right to left. The dynamic deflection of measuring point 2 is estimated by the LDM and the GECS. Figure 9 shows the comparison results of estimated dynamic deflection in time domain, from which it can be found that the estimating results by the three-point deflection estimation method are close to those of the LDM. Therefore, the proposed method is very suited for tracing the dynamic response of the bridge.

**Figure 10.**
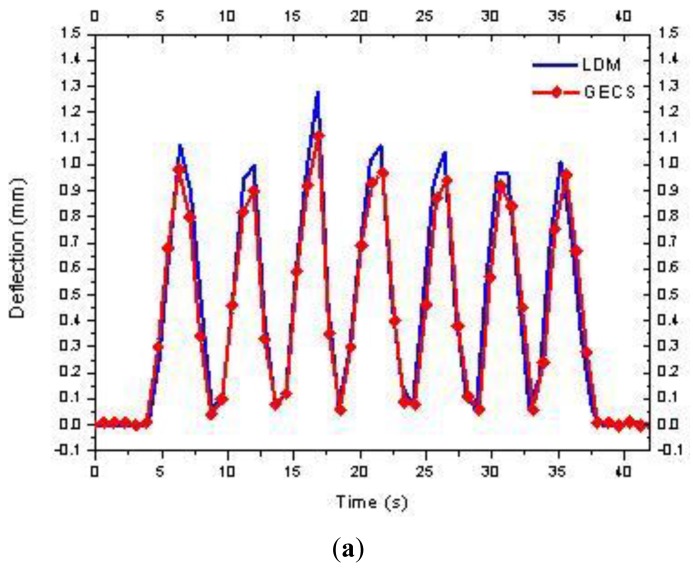

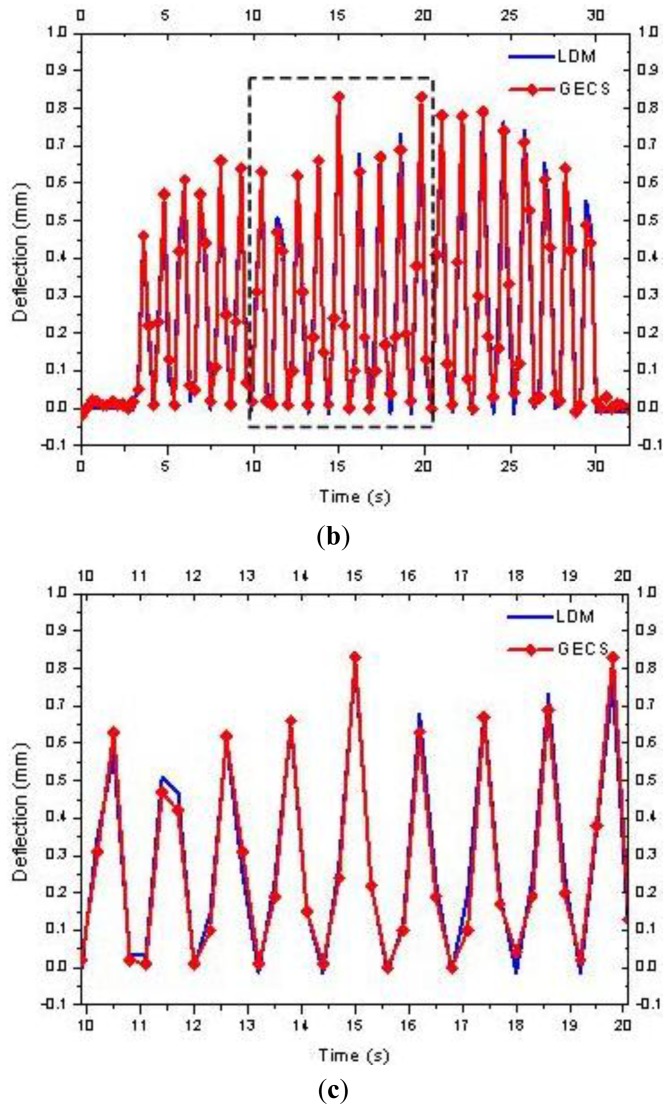
Results of vibration test: (**a**) f = 0.2 Hz, (**b**) and (**c**) f = 1 Hz.

In the vibration tests, frequency components of the bridge model are very complex, and among them, vibration displacement under the natural vibration frequency is the key research object rather than that of the high frequency vibration. Thus, the type of the estimating object in the bridge vibration is also the dynamic displacement, that is to say, the proposed three-point method is also ideal for dynamic vibration tests. For the laboratory model in this paper, a random vibration force is imposed on the beam, and the measuring point 2 of which the dynamic deflection variation range is more obvious than the others is taken as the observation point. Figure 10 shows the comparison results of deflection time history curves of measuring point 2 estimated by the two methods in vibration tests.

It also can be seen that the two curves are fitted well, which indicates that the proposed three-point method is also appropriate for dynamic estimations. Plenty of static and dynamic experimental results demonstrate that the three-point method presents comprehensive reliability.

## 5. Conclusions

A reliable three-point deflection estimation method using the GECS is proposed in this paper. This method takes the real spatial positions of the measuring points as relative reference points of each other directly without using any other auxiliary referenced points, which offers great convenience for bridge deflection estimation. The accurate relative deflection estimated by the GECS of each measuring unit gives security for the calculation of absolute deflections. The selected displacement sensor GECS is not susceptible to the velocity of displacement variation, based on which both static and dynamic deflections can be calculated from the relative deflections directly without any correction approaches. As an inductive sensor, moreover, the selected GECS is waterproof and dustproof in principle, thus it can work under bad weather conditions, which makes it very suitable for long-term monitoring. Both static and dynamic experiments have been carried out on a simple beam laboratory bridge model, and the experimental results indicate that the proposed method is adequate for deflection estimation with great precision, which show the effectiveness of the three-point method and offers an alternative way for bridge deflection estimation. For the purpose of enhancing its efficiency, wireless data transmission technology will be introduced in this estimating system in the future.

## Figures and Tables

**Table 1.** Static deflection estimating results.

**Weight of the load**	**1.6 kg**			**2.6 kg**			**3.6 kg**		
Measuring point	1	2	3	1	2	3	1	2	3
Relative deflection (mm)	0.13	0.21	0.07	0.14	0.30	0.10	0.21	0.38	0.11
Calculated deflection (mm)	0.44	0.62	0.38	0.56	0.84	0.52	0.75	1.08	0.65
Dial gauges (mm)	0.46	0.62	0.39	0.58	0.82	0.51	0.78	1.08	0.68
Relative error (%)	−4.3	0	−2.6	−3.4	2.4	1.9	−3.8	0	−4.4
